# Supplementary material for: ChronoRoot: High-throughput phenotyping by deep segmentation networks reveals novel temporal parameters of plant root system architecture
Source: Gigascience. 2021 Jul 20;10(7):giab052. doi: 10.1093/gigascience/giab052 (PMC8290196; doi:10.1093/gigascience/giab052)
Supplement: giab052_Response_to_Reviewer_Comments_Original_Submission [file giab052_response_to_reviewer_comments_original_submission.pdf]

Author's Response To Reviewer Comments

Close

Answer to reviewers

Dear GigasScience Editor,

We would like to thank you for your positive feedback and the reviewers for their comments. In what follows, we provide a point-by-point response to the observations raised by the reviewers.

Reviewer #1:

R1: As above, the discussion of prior art is light, and it is very relevant for a paper like this proposing a new pipeline. Your image capture setup based on 3D printed parts looks interesting, but how does it compare to existing offerings such as [https://onlinelibrary.wiley.com/doi/pdfdirect/10.1111/tpj.13472] or [https://royalsocietypublishing.org/doi/full/10.1098/rstb.2011.0291]?

→ In contrast to the root imaging system developed by Wells and coworkers (2012), here we designed a self-contained module based on low-cost and largely available consumables in comparison to industrial hardware, which is remarkably more expensive. In addition, with ChronoRoot we overcame the need for robot movement and we decreased the complexity of the device which no longer needs to be fixed into a growth chamber. Our modules can be located easily in already existing facilities without major modifications or permanent movement. The number of modules to be built and used will only depend on the available space and the experimental design (e.g. a few modules for the characterization of given genotypes vs. multiple units for GWAS approaches using tens to hundreds of plant accessions). We collect the same type of image as in this reference with a lighter and modular hardware design.

Regarding the Phenotiki device, we used the same kind of approach based on low-cost available hardware and 3D printing. Phenotiki was first designed for the phenotypic characterization of aerial organs of the plant. In contrast, ChronoRoot was conceived specifically to allow root high-throughput phenotyping. To this end, ChronoRoot includes plates support and backlighting allowing the monitoring of root growth independently of the growth chamber conditions (as also proposed by Wells and coworkers). More recently, the Phenotiki sensor interface was applied to the analysis of root growth using Rizoboxes in Bontpart et al (2020), although the images captured by their device together with the proposed conventional image analysis pipeline can only account for global root traits (like total root area, convex hull area, total root length, etc). Thus, while the approach proposed in Bontpart et al (2020) focuses on temporal extraction of global RSA traits, ChronoRoot allows for a more fine-grained high-throughput temporal phenotyping, for example making it possible to distinguish between main and lateral roots.

This is now further discussed in the second paragraph of the Discussion section (page 8, marked in blue).

R1: In terms of image analysis, you cite most of the relevant prior work in this area, but very briefly. Roottrace captures root traits over time, does your approach offer a better alternative to this? What are the benefits of using your CNN segmentation over e.g. RootNav 2? Other tools such as GiaRoots, EZ Rhizo, Win Rhizo etc. utilise a pipeline in which morphology and skeletonisation are applied after segmentation (thresholding rather than CNN-based). Does your work offer superior performance to these? I would imagine so, since this is CNN based, but this is not described. I think you can address these questions, and doing so would increase the impact of your paper.

→ We thank the reviewer for pointing this out. Here we highlight the main differences with existing RootTrace (French et al 2009, Naeem et al 2011), GiaRoots (Galkovskyi et al, 2012), EZ Rhizo (Armengaud et al, 2009), Win Rhizo, BRAT (Slovak et al, 2014) and RootNav 2 (Yasrab et al, 2019), and we include this discussion in the main manuscript (Discussion section, in blue).

Similarly to our work, RootTrace also focuses on high-throughput analyses of root growth. However, it employs traditional image processing and tracking techniques, resulting in a program that can only extract MR length and count the number of emerged LRs. On the contrary, our model relies on deep networks producing a detailed segmentation of the RSA which is then classified into MR and LR, allowing for fine-grained measurements like the total length of the LRs, which is not provided by RootTrace. Other tools such as GiaRoots (Galkovskyi et al, 2012) and EZ Rhizo (Armengaud et al, 2009) employ simple threshold strategies for root segmentation. In contrast to ChronoRoot, these tools fall short at handling segmentation problems emerging from drops due to water condensation, they require manual human calibration and do not take advantage of the redundancy provided by the temporal resolution of the high-throughput videos to filter out spurious segmentations. Another alternative tool is Win Rhizo, a commercial and non-open source tool designed to work with images captured with high resolution desktop optical scanners. Such a requirement makes it virtually impossible to capture high-throughput temporal sequences of growing plants. On the contrary, ChronoRoot is open-source and designed to work with low-cost cameras. Another option is BRAT (Slovak et al, 2014), designed for high-throughput phenotyping of root growth and development. The main disadvantage of BRAT is that it can only handle early root growth, and does not provide measurements for LRs.

The previously discussed methods are mostly based on conventional image processing approaches and extract a limited number of RSA features. Closest to our work is the recent RootNav 2 (Yasrab et al, 2019), which is also based on deep learning models and provides fine-grained metrics distinguishing between MR and LRs. However, RootNav 2 does not exploit the redundancy provided by the temporal resolution and follows a different architectural design, which makes ground truth annotations more difficult to obtain, preventing us from training the model with our dataset. Compared to ChronoRoot, RootNav employs a more complex neural network architecture with 2 output paths: the first one is used to predict root segmentation masks (differentiating between MR and LRs) while the second one produces heat maps associated with root tips. This design choice requires the ground truth annotations to be composed of 3 parts: (1) MR pixel level annotations, (2) LR pixel level annotations and (3) root tip annotations. Conversely, ChronoRoot just requires binary segmentation maps (background vs foreground root) for training, since the MR and LR labeling is performed after segmentation following a deep first search approach on the skeletonized binary segmentation. Thus, our dataset is just composed of images with foreground/background pixel level annotations, which is not enough for training the RootNav 2 model. It is also worth mentioning that we tried to run the pre-trained RootNav Arabidopsis model available online, but it failed to segment our images. We believe this is due to the domain shift introduced by the different acquisition conditions and devices.

We have now substantially extended the prior work discussion in the main manuscript. Please see the 4th and 5th paragraph of the Discussion section in Page 8 (highlighted in blue).

R1: Your use of an ensemble network for segmentation is interesting, and seems to show good performance - Dice and Hausdorff are good metrics to use. This looks like a novel contribution to me. However you have not provided any images showing segmentation output (aside from small ones in Figure 1). This component is key to the accuracy of the proceeding image analysis steps such as skeletonisation and graph extraction.

→ We have now included a new figure (Figure 6) showing qualitative results for the RSA segmented using the proposed and benchmarked deep segmentation networks, as well as the ensemble of models.

R1: How does the network perform as the root systems get more complex as the plants get older?

→ There are two main challenges that appear when plants get older: (1) mature plants have heavier aerial organs, which tend to fall down and occlude the roots; and (2) when plants get older, multiple crosses between the MR and LRs appear, making the distinction between them more challenging. First, point 1 (root occlusion due to aerial parts falling down) is the main problem affecting the performance of the segmentation network, since it is really difficult to segment root parts which are hidden behind a leaf. That is why, when leaves fall too early, we directly remove the individuals from the experiment to avoid measurement issues. Second, the main impact of point 2 is not related to the segmentation step, since the network's output is binary (root vs background). However, complex RSA exhibited by older plants are difficult to classify in MR/LR. That is why we restricted our experiments to 14 days, which was long enough to find discriminative temporal phenotypes in the explored scenario.

R1: This is also a challenge for most other approaches in previous work. Can we reliably expect lateral root length to remain an accurate measure? You have a large spread in your data in Figure 2, is this

caused by natural variability in the plant, or noise introduced when skeletonising complex root systems? Along similar lines, you have not shown any examples of the graphs extracted, as such it is difficult for the reader to know how accurate and robust they may expect this step to be.

→ As stated before, we restricted the experiments to 14 days since it was a long enough period for our analysis, while it keeps a manageable complexity for the RSA. Regarding lateral root length, please note that even though we distinguish between MR and LR length, when computing the LR traits, we consider the total LR length, not the individual length for every LR. This aggregation step makes the feature more robust to potential problems that may emerge during graph construction (e.g. misidentification of cross-points between lateral roots will not affect this parameter, which is computed by looking at the LR as a whole). We believe the spread identified by the reviewer is mainly due to natural variability.

We have now included a figure (Figure 7) with extracted graphs to better illustrate the expected outcomes of our model for RSAs exhibiting different levels of complexity.

R1: The majority of your pipeline is automatic, which is of course a good benefit for anyone using your system for high-throughput analysis. I was somewhat confused by the inclusion of a manual user ROI procedure to separate the plants. Once segmented, is there not some process that can be applied to separate each plant automatically? E.g. based on the size of connected components? If not, what is the limitation of the segmentation that is preventing this?

→ We included the manual user ROI step for two main reasons: usability and correctness of the results.

- Usability: First, it allows biologists to choose which individuals are going to be processed and included in the quantitative RSA traits computation. In most cases, certain individuals need to be excluded for multiple reasons (e.g. plants not growing or falling down quickly). In other cases, individuals of multiple genotypes may be present on the same plate, and only a few plants will be analyzed each time (first one genotype, then the other). By allowing biologists to choose the ROI corresponding to a single individual, we can filter out problematic and undesired plants.

- Correctness: When two plants are growing on the same plate, it could happen that they cross with each other. Note that in order to generate the ROI automatically, it would be necessary to use the segmentation from the last frames of the time series, since those indicate the full extent covered by the plant. In cases where the plants cross, this may result in an erroneous single big ROI being selected.

Note that in case the user is interested in processing absolutely all the plants, a simple connected components algorithm could be used to automatically choose the individuals.

R1: Your temporal consistency is an interesting approach, but without quantitative or qualitative data it is difficult to judge the success. Is an average of two time steps a true reflection of the segmentation? Are the two time steps sufficiently close that the plant hasn't grown much, and as such this represents more of a noise removal step?

→ We thank the reviewer for this comment. We now realize that we have not provided all the details about the temporal averaging in the original manuscript, so we are incorporating them in the revised version. The temporal averaging step is a weighted average between the current segmentation and an accumulation of the previous ones, which helps to avoid losing parts of the root due to droplets or other type of occlusion. The idea is to use the root segmentation masks obtained in previous time steps to correct for potentially missing root segments. The current segmentation value st for a pixel is smoothed by $a_t = s_t + 0.9 a_{t-1}$. Note that the accumulation $a_{t-1} = s_{t-1} + 0.9 a_{t-2}$, and substituting it in the first equation we have $a_t = s_t + 0.9 (s_{t-1} + 0.9 a_{t-2}) = s_t + 0.9 s_{t-1} + 0.81 a_{t-2}$. As it can be seen, the current value of the segmentation takes into account all the previous ones, with weights that are higher for the most recent in time: $a_t = s_t + 0.9 s_{t-1} + 0.81 s_{t-2} + 0.73 s_{t-3} + 0.65 s_{t-4} + 0.59 s_{t-5}$…. This is now discussed in the section "Graph generation and temporal consistency improvement". For qualitative results, please see the new Figure 6 included in the manuscript.

R1: Your description of the graph extraction step could be a little more detailed. What is the process for assigning labels to the seed, primary and lateral root tips? What graph matching algorithm do you use?
→ The graph extraction step starts by first skeletonizing the binary dense segmentation masks, which provide an unlabeled graph. We then run a deep first search (DFS) algorithm in order to label the bifurcation and end nodes of the unlabeled root graph given by the skeletonized binary segmentation.

We use the DFS algorithm, starting from a seed that can be automatically chosen as the top pixel in the plant ROI or manually specified. For assigning labels to the MR, we work based on the assumption that in early growing stages, there will only be a MR with seed (top pixel) and tip (bottom pixel). We then use nearest neighbours for matching the node graphs in the succeeding iterations. As more nodes appear deviating from the MR, they will be added as bifurcation (more than one neighbour) or lateral root tip (one neighbour, different from the MR tip). In case that one LR collides with the main root or another LR, the tip will still be a tip because of the matching process. Following this procedure, labels are assigned for the seed, main root tip, bifurcation and lateral root tip nodes. Node graph matching based on a nearest neighbor criterion was performed between the labeled nodes of successive graphs in the temporal sequence to track the evolution of the root. These details are now included in the "Graph generation and temporal consistency improvement" section.

R1: What is the biological significance of the crossover point you highlight in Figure 3?

→ The distribution of the root mass into main and lateral roots (and among lateral roots, their length and number) depends largely on the genotype and the environment. In general, the temporal dimension of this architecture is missed when a root system is characterized only in young or older seedlings. Here we propose to determine a novel time-related parameter which reflects the dynamics of root growth by determining how long it takes for the system to be composed of more lateral roots than the main root. This uncovers how by choosing a single time point for root phenotyping we can gain very limited understanding about root development. This is now further discussed in the section "Temporal dimension of traditional and novel RSA parameters".

R1: While my review might read as negative, I am optimistic that many of these issues can be addressed. I also commend the authors on their open source approach to this, including plans and details of the image capture setup. Does the consistency of imaging afforded by this setup mean that it's likely your CNN would work in new installations elsewhere without retraining? This would be a good benefit to highlight.

→ Your feedback is valuable and we welcome it! Exactly, for anybody installing the system and using the same imaging setup they will be able to use this software for Arabidopsis thaliana without retraining. This is now mentioned in the Potential Implications section.

Reviewer #2

Major comment

Whereas the authors provide quantitative evaluation for different CNN architectures in a dense root segmentation task, it is not clear to me whether this renders ChronoRoot preferable to alternative methods of measuring root system growth dynamics. The authors allude to this point in the Discussion where they state that, "According to Quantitative Plant [31, 32], over 40 image processing softwares are available for root system analysis [33, 29]."

Have the authors compared ChronoRoot to these alternative root system analysis tools? A comparison of ChronoRoot with Quantitative Plant image analysis software tools (https://www.quantitative-plant.org/software) would help to frame the significance of ChronoRoot as an improvement over existing software tools. Consequently, in the Discussion I invite the authors to compare ChronoRoot with existing root system analysis software tools to highlight the advantages of ChronoRoot deep learning-based analysis.

→ Thank you, a similar point was raised by Reviewer 1. We have now highlighted the main differences with existing tools available in RootTrace (French et al 2009, Naeem et al 2011), GiaRoots (Galkovskyi et al, 2012), EZ Rhizo (Armengaud et al, 2009), Win Rhizo, BRAT (Slovak et al, 2014) and RootNav 2 (Yasrab et al, 2019), and we included this discussion in the main manuscript (Discussion section, as suggested by the reviewer).

Similarly to our work, RootTrace also focuses on high-throughput analyses of root growth. However, it employs traditional image processing and tracking techniques, resulting in a program that can only extract MR length and count the number of emerged LRs. On the contrary, our model relies on deep networks producing a detailed segmentation of the RSA which is then classified into MR and LR, allowing for fine-grained measurements like the total length of the LRs, which is not provided by RootTrace.

Other tools such as GiaRoots (Galkovskyi et al, 2012) and EZ Rhizo (Armengaud et al, 2009) employ simple threshold strategies for root segmentation. In contrast to ChronoRoot, these tools fall short at handling segmentation problems emerging from drops due to water condensation, they require manual human calibration and do not take advantage of the redundancy provided by the temporal resolution of the high-throughput videos to filter out spurious segmentations. Another alternative tool is Win Rhizo, a commercial and non-open source tool designed to work with images captured with high resolution desktop optical scanners. Such a requirement makes it virtually impossible to capture high-throughput temporal sequences of growing plants. On the contrary, ChronoRoot is open-source and designed to work with low-cost cameras. Another option is BRAT (Slovak et al, 2014), designed for high-throughput phenotyping of root growth and development. The main disadvantage of BRAT is that it can only handle early root growth, and does not provide measurements for LRs.

The previously discussed methods are mostly based on conventional image processing approaches and extract a limited number of RSA features. Closest to our work is the recent RootNav 2 (Yasrab et al, 2019), which is also based on deep learning models and provides fine-grained metrics distinguishing between MR and LRs. However, RootNav 2 follows a different architectural design, which makes ground truth annotations more difficult to obtain and prevents us from training the model with our dataset. Compared to ChronoRoot, RootNav employs a more complex neural network architecture with 2 output paths: the first one is used to predict root segmentation masks (differentiating between MR and LRs) while the second one produces heat maps associated with root tips. This design choice requires the ground truth annotations to be composed of 3 parts: (1) MR pixel level annotations, (2) LR pixel level annotations and (3) root tip annotations. Conversely, ChronoRoot just requires binary segmentation maps (background vs foreground root) for training, since the MR and LR labeling is performed after segmentation following a deep first search approach on the skeletonized binary segmentation. Thus, our dataset is just composed of images with foreground/background pixel level annotations, which is not enough for training the RootNav 2 model. It is also worth mentioning that we tried to run the pre-trained RootNav Arabidopsis model available online, but it failed to segment our images. We believe this is due to the domain shift introduced by the different acquisition conditions and devices.

As suggested, we have now substantially extended the discussion in the main manuscript. Please see the 4th and 5th paragraph of the Discussion section in Page 8 (highlighted in blue).

Minor comments

R2: An interesting feature of this paper is the inclusion of open hardware, specifically printable components - such as the main board, LED support, camera support, and plate support - are provided as printable STL files. In addition, schematics (SVG format) have also been provided. To enable reuse an Open Source Hardware License, such as CERN 2.0, should be attributed to the open hardware.

→ We thank the reviewer for the recommendation. We have now attributed the CERN 2.0 licence to the open hardware system by including the licence file in the hardware repository. Please note that we have created a new Github repository for the hardware description, which includes the CERN 2.0 Licence (https://github.com/ThomasBlein/ChronoRootModuleHardware).

R2: In addition, the source code used by the ChronoRoot deep learning model and the graph generation procedures are made publicly available on GitHub (https://github.com/ngaggion/ChronoRoot). However, there is no license file associated with this GitHub archive. In the "Availability of source code and requirements" section of the manuscript it states that a GNU GPL license has been attributed to the ChronoRoot deep learning model source code. I request that the authors add a license file to this GitHub archive to encourage reuse.

→ We have now included a GNU GPL licence file in the ChronoRoot repository.

R2: Furthermore, the source code used by the ChronoRoot module controller has been ascribed an OSI-approved CeCILL-2.1 license. However, in the "Availability of source code and requirements" section of the manuscript it states that a GNU GPL license has been attributed to the ChronoRoot module controller source code. I request that the license for this GitHub archive is correctly stated in the manuscript.

→ We thank the reviewer for pointing this out. We have now corrected the manuscript stating that the ChronoRoot module controller has been assigned an OSI-approved CeCILL-2.1 license.

Reviewer #3:

R3: Minor comments:
- Dataset: the authors give a lot of details re the dataset used for the deep learning models. However, these details are machine learning focused (e.g., we have X videos, we annotated Y images, etc.). It's unclear how many plants were used to generate such time series

→ We thank the reviewer for pointing this out. Here we include the missing information for the two datasets used in this study:

- Dataset used to train and validate the deep learning models to benchmark root segmentation: 240 plants in total.
- Use case dataset for plant phenotyping under alternative photoperiods: 25 plants for the CL growth condition, and 25 plants for the LD growth condition.

This information is now included in the "Datasets" section of the main manuscript.

R3: Training/Val set & Experimental dataset: the authors have two datasets: one used for training and validating the models, one to actually applied the trained model in a real-case scenario. Could you please state if these datasets are disjoined (e.g., a plant appearing in the training DOES NOT appear in the other dataset and vice versa). [*]

→ These datasets are disjointed; please see the answer to the major point below where we address this comment.

R3: temporal consistency refinement: as far as I understood from page 10, this step is performed as follows: I take an image at time t and one at time t-1 and I average them. Clearly, the root has grown in between. My question here is: how can you ensure that, by applying this method, you are not also getting rid of new grown material at time t that were not at t-1? Can it happen that you actually remove true positive that were not in the previous frame?

→ We thank the reviewer for this comment, which was also mentioned by R1. We now realize that we have not provided all the details about the temporal averaging in the original manuscript, so we are incorporating them in the revised version. The temporal averaging step is a weighted average between the current segmentation and an accumulation of the previous ones, which helps to avoid losing parts of the root due to droplets or other type of occlusion. The idea is to use the root segmentation masks obtained in previous time steps to correct for potentially missing root segments. The current segmentation value s_t for a pixel is smoothed by $a_t = s_t + 0.9 a_{t-1}$. Note that the accumulation $a_{t-1} = s_{t-1} + 0.9 a_{t-2}$, and substituting it in the first equation we have $a_t = s_t + 0.9 (s_{t-1} + 0.9 a_{t-2}) = s_t + 0.9 s_{t-1} + 0.81 a_{t-2}$. As it can be seen, the current value of the segmentation takes into account all the previous ones, with weights that are higher for the most recent in time: $a_t = s_t + 0.9 s_{t-1} + 0.81 s_{t-2} + 0.73 s_{t-3} + 0.65 s_{t-4} + 0.59 s_{t-5}$…. Assigning higher weights to the recent frames mitigates the issue pointed by the reviewer. This is now discussed in the section "Graph generation and temporal consistency improvement". For qualitative results, please see the new Figure 6 included in the manuscript.

R3: Analysis section:
"Our model takes a sequence of images as input and outputs a labeled graph for each frame" I don't think this statement is correct. In my mind (and also according to what the authors meant), a model is referred to the deep learning models, right? As such, the output of such models is not a labeled graph, but it's a segmentation mask. Did the author mean to say "Our framework takes a sequence [...]"?

→ Thanks for pointing this out. We have now changed this in the main manuscript stating that "Our framework takes a sequence ...".

R3: References to state-of-the-art methods (e.g., unet) are missing in this section

→ We have now revised the section and included the missing references.

R3: Results of table 1 comes before the authors introduce what CRF is and what it's for. In fact, I looked at the table as I was reading the paper and I was confused by the presence of Table 1. I suggest the authors to present the results AFTER they have introduced all the things contained in table 1.

→ We have now moved the table to the bottom part of the page, so that it appears after the first part of the Analyses section, where the most important components of ChronoRoot necessary to understand the table are discussed.

R3: "As shown in Figure 1, we apply several post-processing steps after segmentation" I don't think Fig 1 actually shows any post-processing. After step 4 (deep net), there is ROI selection and multi-class labeling

→ We have changed this sentence to reflect the reviewer's comment removing "As shown in Figure 1".

R3: Fig 5(c): Frequency is misspelled

→ We corrected the typo in Figure 5.

R3: I am not a plant biologist and I apologise in advance if my doubt here sounds naive. At the end, I could not understand what the reason behind the FFT analysis. At the end, what is this analysis telling me (please explain this in lay terms as I hardly got the message written in the text).

→ The FFT analysis helps to better understand the differences in growth patterns exhibited by alternative growth conditions. Fourier transform decomposes functions depending on time into functions depending on frequency. In other words, the Fourier transform of a given function describes how much of any given frequency is present in the original signal. When comparing growth speed signals, analysing their Fourier spectrum helps us to see how much this signal correlates with particular oscillation frequencies. For example, if high Fourier coefficients are associated with the frequency 1/24h, it means that the plant tends to change its growth speed following a daily oscillation. From a biological perspective, this is related to many processes oscillating during the day, what is known as "circadian rhythm" (similarly, the frequency 1/12h is known in biology as "ultradian rhythm"). If two signals have big differences in the Fourier components associated with frequency 1/24h, it means that they are not following the same oscillation pattern. For instance, here we have shown that by growing plants in continuous light, the circadian rhythm is impaired, in agreement with the difference in the frequency 1/24h observed comparing with long day-treated plants. Plants affected in the perception of day-night rhythm will behave differently in terms of growth variation, which will be highlighted by frequency analysis. We have now clarified this point in the section "Novel speed-based parameters derived from temporal phenotyping" of the revised manuscript (indicated in blue).

R3: CRF: on page 9, the authors say the parameters for the CRF are theta=5 and theta=3. Is it correct that the same parameter has two values?

→ Thanks for pointing this out. The correct statement should be \theta_\alpha = 5 and theta_\beta = 3. We have now changed this in the manuscript.

R3: When the authors derive the graph representation of the plants, how do they deal with discontinuities arising from the segmentation process? In my experience, some times lateral roots exhibit discontinuity near the branching point.

→ The time consistency refinement accounts for this issue, especially when small discontinuities appear, since those are filled thanks to the information provided by the temporal redundancy.

R3: The authors used a dicot plant (arabidopsis thaliana). Could please the authors comment (only here, they don't need to change the main paper to answer to this question) how their work would apply for monocot (e.g., barley) plants? In my experience, lateral roots of barley are rather thin and hard to be segmented.

→ To date, we have not tested our model in monocot plants. However, if enough annotations are manually constructed, we could re-train the proposed network to segment monocot plants. As the reviewer is suggesting, when roots are very thin, the model may tend to under-segment them. A potential alternative to deal with this problem would be to avoid the dense segmentation step, and pose the RSA delineation problem as an end-to-end image to graph extraction problem, where the RSA graph is directly considered as the output of the neural network. Following this approach would avoid the dense segmentation stage, which is most affected by the thin roots. Additionally, monocot plants include additional embryonic and post-embryonic roots, i.e. crown roots, seminal roots, etc, some of which are

even shoot-born roots. Therefore, characterizing the root system of monocots may represent a more challenging task, requiring further annotations and training.

R3: Major point:

Picking up my second minor comment (the one marked with a [*]), I have this doubt. During the training, it is not clear how the dataset was split between training/ validation (if used)/test set. Are they treating each image individually? Or they are treating each time series (videos as they call) individually? This is important to be clarified for the following reasons:
- Images treated individually: in this case, it can happen that the image of a plant at time t can appear in the training set, while another frame of the same plant (let's say t+2) happens to be in the testing set. If this is the case, I think this is wrong because the testing set contains images that the network has already seen (although at a different development stage). In my personal experience, I've got rather biased (too good to be true!) results when images of a time series are used independently to each other.
- Time series used individually: if n time series are used for training and m for testing (and they are disjoined), then this is the correct procedure.
Therefore, I ask the authors to clarify which of the two protocols they used. In the case they treated each image independently, I highly recommend to run all the training and testing again treating each time series as a whole.

→ We thank the reviewer for this comment. Let us clarify how the datasets where constructed and used:

- Dataset 1: Dataset used to train and validate the deep learning models for root segmentation. This is the dataset used to evaluate the quality of the segmentation networks, i.e. to compute the results shown in Table 1. When constructing the training/validation/test splits for these experiments, we considered time series individually to ensure that the quantitative results are not biased, as suggested by the reviewer. The results for dice, hausdorff and recall metrics were reported using the hold-out set of videos.

- Dataset 2: Use case dataset for plant phenotyping under alternative photoperiods. This dataset was used to showcase how ChronoRoot can be used to extract temporal phenotypes, but not to evaluate the quality of the segmentation network. The model used to segment this dataset had been trained using a random split of annotated images. In any case, given your comment and to ensure that there is no overlapping between the plants used to train the model and those used in the downstream phenotyping analysis, we have re-trained it again making sure that time series are treated individually and updated all the Figures (2 to 5) accordingly. The same trends discussed in the original manuscript are still observed.

We have now clarified this point in the dataset description section.

Close
