# Supplementary material for: ChronoRoot: High-throughput phenotyping by deep segmentation networks reveals novel temporal parameters of plant root system architecture
Source: Gigascience. 2021 Jul 20;10(7):giab052. doi: 10.1093/gigascience/giab052 (PMC8290196; doi:10.1093/gigascience/giab052)
Supplement: giab052_Response_to_Reviewer_Comments_Revision_1 [file giab052_response_to_reviewer_comments_revision_1.pdf]

Author's Response To Reviewer Comments

Close

We thank the editor and the reviewer for their comments. Here we include the minor changes we did to the manuscript in this final submission:
- ORCID profiles links were added as requested
- "Deep first search", was changed as "depth first search" in all document, as noticed by reviewer 1
- A reference to the GigaDB repository was added, alongside this sentence "Snapshots of our code and other data further supporting this work are openly available in the GigaScience Repository, GigaDB[60]." at the end of "Availability of supporting data and materials" section
- "11 of those images were annotated by an expert biologist" was changed to "11 of those videos were annotated by an expert biologist" in Dataset section.

Close
