# Supplementary material for: ChronoRoot: High-throughput phenotyping by deep segmentation networks reveals novel temporal parameters of plant root system architecture
Source: Gigascience. 2021 Jul 20;10(7):giab052. doi: 10.1093/gigascience/giab052 (PMC8290196; doi:10.1093/gigascience/giab052)
Supplement: giab052_Response_to_Reviewer_Comments_Revision_2 [file giab052_response_to_reviewer_comments_revision_2.pdf]

Author's Response To Reviewer Comments

Close

We have now added the missing reference [60].

Close
