# Supplementary material for: ChronoRoot: High-throughput phenotyping by deep segmentation networks reveals novel temporal parameters of plant root system architecture
Source: Gigascience. 2021 Jul 20;10(7):giab052. doi: 10.1093/gigascience/giab052 (PMC8290196; doi:10.1093/gigascience/giab052)
Supplement: giab052_Reviewer_1_Report_Original_Submission — Michael Pound -- 1/26/2021 Reviewed [file giab052_reviewer_1_report_original_submission.pdf]

Reviewer Report

Title: ChronoRoot: High-throughput phenotyping by deep segmentation networks reveals novel temporal parameters of plant root system architecture

Version: Original Submission **Date:** 1/26/2021

Reviewer name: Michael Pound

Reviewer Comments to Author:

The authors present a system to grow, image, segment and analyse root systems grown on gel plates. The system comprises a number of features, first a 3D printed imaging setup powered by raspberry pi cameras collects image sequences of seedlings growing over time. These are processed by a deep network to provide segmented images, which are processed using techniques such as skeletonisation in order to derive a root graph. These graphs are examined over time and results are reported on a study showing the different growth rates of Arabidopsis grown under different photoperiods.
The manuscript is well written, the text is easy to follow and the figures are clear. The results suggest that differences in e.g. growth conditions are indeed represented by these temporal traits. In its current form my main concern for the paper is that it is somewhat light on a discussion of existing techniques in this area. This makes it challenging for a reader to gauge novely, importance, and whether or not to utilise this work over some other work. Previous works are cited, but rarely are the strengths and weaknesses discussed against this new system, and details on these works are light. Root imaging is a field that has been producing software tools for over 10 years, there are dozens of software packages, some specifically written to handle arabidopsis plate images. These should be discussed, what new ability results from your pipeline? There is also not enough information to guage the efficacy of the segmentation and graph extraction steps. The majority of the results presented are end of process measurements, but of course there are many things between the image capture and this step. Here are some detailed points that I feel must be addressed:
- As above, the discussion of prior art is light, and it is very relevant for a paper like this proposing a new pipeline. Your image capture setup based on 3D printed parts looks interesting, but how does it compare to existing offerings such as [https://onlinelibrary.wiley.com/doi/pdfdirect/10.1111/tpj.13472] or [https://royalsocietypublishing.org/doi/full/10.1098/rstb.2011.0291]? In terms of image analysis, you cite most of the relevant prior work in this area, but very briefly. Roottrace captures root traits over time, does your approach offer a better alternative to this? What are the benefits of using your CNN segmentation over e.g. RootNav 2? Other tools such as GiaRoots, EZ Rhizo, Win Rhizo etc. utilise a pipeline in which morphology and skeletonisation are applied after segmentation (thresholding rather than CNN-based). Does your work offer superior performance to these? I would imagine so, since this is CNN based, but this is not described. I think you can address these questions, and doing so would increase the impact of your paper.
- Your use of an ensemble network for segmentation is interesting, and seems to show good performance - Dice and Hausdorff are good metrics to use. This looks like a novel contribution to me. However you have not provided any images showing segmentation output (aside from small ones in

Figure 1). This component is key to the accuracy of the proceeding image analysis steps such as skeletonisation and graph extraction. How does the network perform as the root systems get more complex as the plants get older? This is also a challenge for most other approaches in previous work. Can we reliably expect lateral root length to remain an accurate measure? You have a large spread in your data in Figure 2, is this caused by natural variability in the plant, or noise introduced when skeletonising complex root systems? Along similar lines, you have not shown any examples of the graphs extracted, as such it is difficult for the reader to know how accurate and robust they may expect this step to be.

- The majority of your pipeline is automatic, which is of course a good benefit for anyone using your system for high-throughput analysis. I was somewhat confused by the inclusion of a manual user ROI proceedure to separate the plants. Once segmented, is there not some process that can be applied to separate each plant automatically? E.g. based on the size of connected components? If not, what is the limitation of the segmentation that is preventing this?
- Your temporal consistency is an interesting approach, but without quantitative or qualitative data it is difficult to judge the success. Is an average of two time steps a true reflection of the segmentation? Are the two time steps sufficiently close that the plant hasn't grown much, and as such this represents more of a noise removal step?
- Your description of the graph extraction step could be a little more detailed. What is the process for assigning labels to the seed, primary and lateral root tips? What graph matching algorithm do you use?
- What is the biological significance of the crossover point you highlight in Figure 3?

While my review might read as negative, I am optimistic that many of these issues can be addressed. I also commend the authors on their open source approach to this, including plans and details of the image capture setup. Does the consistency of imaging afforded by this setup mean that it's likely your CNN would work in new installations elsewhere without retraining? This would be a good benefit to highlight.

Methods

Are the methods appropriate to the aims of the study, are they well described, and are necessary controls included? Choose an item.

Conclusions

Are the conclusions adequately supported by the data shown? Choose an item.

Reporting Standards

Does the manuscript adhere to the journal's guidelines on minimum standards of reporting? Choose an item.

Choose an item.

Statistics

Are you able to assess all statistics in the manuscript, including the appropriateness of statistical tests used? Choose an item.

Quality of Written English

Please indicate the quality of language in the manuscript: Choose an item.

Declaration of Competing Interests

Please complete a declaration of competing interests, considering the following questions:

- Have you in the past five years received reimbursements, fees, funding, or salary from an organisation that may in any way gain or lose financially from the publication of this manuscript, either now or in the future?
- Do you hold any stocks or shares in an organisation that may in any way gain or lose financially from the publication of this manuscript, either now or in the future?
- Do you hold or are you currently applying for any patents relating to the content of the manuscript?
- Have you received reimbursements, fees, funding, or salary from an organization that holds or has applied for patents relating to the content of the manuscript?
- Do you have any other financial competing interests?
- Do you have any non-financial competing interests in relation to this paper?

If you can answer no to all of the above, write 'I declare that I have no competing interests' below. If your reply is yes to any, please give details below.

I declare that I have no competing interests

I agree to the open peer review policy of the journal. I understand that my name will be included on my report to the authors and, if the manuscript is accepted for publication, my named report including any attachments I upload will be posted on the website along with the authors' responses. I agree for my report to be made available under an Open Access Creative Commons CC-BY license (http://creativecommons.org/licenses/by/4.0/). I understand that any comments which I do not wish to be included in my named report can be included as confidential comments to the editors, which will not be published.

Choose an item.

To further support our reviewers, we have joined with Publons, where you can gain additional credit to further highlight your hard work (see: https://publons.com/journal/530/gigascience). On publication of this paper, your review will be automatically added to Publons, you can then choose whether or not to claim your Publons credit. I understand this statement.

Yes Choose an item.
