# Supplementary material for: ChronoRoot: High-throughput phenotyping by deep segmentation networks reveals novel temporal parameters of plant root system architecture
Source: Gigascience. 2021 Jul 20;10(7):giab052. doi: 10.1093/gigascience/giab052 (PMC8290196; doi:10.1093/gigascience/giab052)
Supplement: giab052_Reviewer_2_Report_Original_Submission — Chris Armit -- 1/28/2021 Reviewed [file giab052_reviewer_2_report_original_submission.pdf]

Reviewer Report

Title: ChronoRoot: High-throughput phenotyping by deep segmentation networks reveals novel temporal parameters of plant root system architecture

Version: Original Submission **Date:** 1/28/2021

Reviewer name: Chris Armit

Reviewer Comments to Author:

In this Research Article, the authors report on ChronoRoot, which is a high-throughput deep learning approach to facilitate automated phenotyping of Arabidopsis root morphology. ChronoRoot involves image capture, root segmentation, selection of regions of interest, multi-class labelling and graph extraction. This process renders a skeleton of the plant root, and pixels in the skeleton are identified as belonging to the main root or lateral root. Temporal differences in skeleton morphology can then be used as a means of measuring root system growth dynamics over time. In support of the ChronoRoot analysis, the authors provide Use Case Datasets and a Labeled Dataset. The Labeled Dataset contains both Ground Truth Train and Test image data in 16-bit NIfTI format, and these images can be opened using Fiji / Image J.

Major comment

Whereas the authors provide quantitative evaluation for different CNN architectures in a dense root segmentation task, it is not clear to me whether this renders ChronoRoot preferable to alternative methods of measuring root system growth dynamics. The authors allude to this point in the Discussion where they state that, "According to Quantitative Plant [31, 32], over 40 image processing softwares are available for root system analysis [33, 29]."

Have the authors compared ChronoRoot to these alternative root system analysis tools? A comparison of ChronoRoot with Quantitative Plant image analysis software tools (https://www.quantitative-plant.org/software) would help to frame the significance of ChronoRoot as an improvement over existing software tools. Consequently, in the Discussion I invite the authors to compare ChronoRoot with existing root system analysis software tools to highlight the advantages of ChronoRoot deep learning-based analysis.

Minor comments

An interesting feature of this paper is the inclusion of open hardware, specifically printable components - such as the main board, LED support, camera support, and plate support - are provided as printable STL files. In addition, schematics (SVG format) have also been provided. To enable reuse an Open Source Hardware License, such as CERN 2.0, should be attributed to the open hardware.

In addition, the source code used by the ChronoRoot deep learning model and the graph generation procedures are made publicly available on GitHub (https://github.com/ngaggion/ChronoRoot).

However, there is no license file associated with this GitHub archive. In the "Availability of source code and requirements" section of the manuscript it states that a GNU GPL license has been attributed to the ChronoRoot deep learning model source code. I request that the authors add a license file to this GitHub archive to encourage reuse.

Furthermore, the source code used by the ChronoRoot module controller has been ascribed an OSI-approved CeCILL-2.1 license. However, in the "Availability of source code and requirements" section of the manuscript it states that a GNU GPL license has been attributed to the ChronoRoot module controller source code. I request that the license for this GitHub archive is correctly stated in the manuscript.

Methods

Are the methods appropriate to the aims of the study, are they well described, and are necessary controls included? Choose an item.

Conclusions

Are the conclusions adequately supported by the data shown? Choose an item.

Reporting Standards

Does the manuscript adhere to the journal's guidelines on [minimum standards of reporting?]() Choose an item.

Choose an item.

Statistics

Are you able to assess all statistics in the manuscript, including the appropriateness of statistical tests used? Choose an item.

Quality of Written English

Please indicate the quality of language in the manuscript: Choose an item.

Declaration of Competing Interests

Please complete a declaration of competing interests, considering the following questions:

- Have you in the past five years received reimbursements, fees, funding, or salary from an organisation that may in any way gain or lose financially from the publication of this manuscript, either now or in the future?
- Do you hold any stocks or shares in an organisation that may in any way gain or lose financially from the publication of this manuscript, either now or in the future?
- Do you hold or are you currently applying for any patents relating to the content of the manuscript?
- Have you received reimbursements, fees, funding, or salary from an organization that holds or has applied for patents relating to the content of the manuscript?

- Do you have any other financial competing interests?
- Do you have any non-financial competing interests in relation to this paper?

If you can answer no to all of the above, write 'I declare that I have no competing interests' below. If your reply is yes to any, please give details below.

I declare that I have no competing interests.

I agree to the open peer review policy of the journal. I understand that my name will be included on my report to the authors and, if the manuscript is accepted for publication, my named report including any attachments I upload will be posted on the website along with the authors' responses. I agree for my report to be made available under an Open Access Creative Commons CC-BY license (http://creativecommons.org/licenses/by/4.0/). I understand that any comments which I do not wish to be included in my named report can be included as confidential comments to the editors, which will not be published.

Choose an item.

To further support our reviewers, we have joined with Publons, where you can gain additional credit to further highlight your hard work (see: https://publons.com/journal/530/gigascience). On publication of this paper, your review will be automatically added to Publons, you can then choose whether or not to claim your Publons credit. I understand this statement.

Yes Choose an item.
