# Supplementary material for: ChronoRoot: High-throughput phenotyping by deep segmentation networks reveals novel temporal parameters of plant root system architecture
Source: Gigascience. 2021 Jul 20;10(7):giab052. doi: 10.1093/gigascience/giab052 (PMC8290196; doi:10.1093/gigascience/giab052)
Supplement: giab052_Reviewer_2_Report_Revision_1 — Chris Armit -- 6/15/2021 Reviewed [file giab052_reviewer_2_report_revision_1.pdf]

Reviewer Report

Title: ChronoRoot: High-throughput phenotyping by deep segmentation networks reveals novel temporal parameters of plant root system architecture

Version: Revision 1 **Date:** 6/15/2021

Reviewer name: Chris Armit

Reviewer Comments to Author:

I thank the authors for addressing my comments. The authors have now revised the Discussion to include more detail on how ChronoRoot compares to alternative root system analysis tools, such as RootTrace that does not use deep networks, but also GiaRoots and EZ Rhizo which require manual intervention to overcome segmentation artefacts. I think this is a very helpful addition and highlights the utility of the ChronoRoot high-throughput deep learning approach for automated phenotyping of root morphology.

In addition, the authors have now addressed my comments relating to license issues. Specifically the authors have now attributed an OSI-approved GNU GPL license to the ChronoRoot software GitHub repository, and a CERN 2.0 licence to the open hardware system ChronoRoot module (3D print and laser cut) GitHub repository. These important additions ensure that the manuscript adheres to the GigaScience guidelines on minimum standards of reporting, and encourage reuse of both the ChronoRoot software and supporting hardware.

I recommend this Research Article for publication in GigaScience.

Methods

Are the methods appropriate to the aims of the study, are they well described, and are necessary controls included? Choose an item.

Conclusions

Are the conclusions adequately supported by the data shown? Choose an item.

Reporting Standards

Does the manuscript adhere to the journal's guidelines on minimum standards of reporting? Choose an item.

Choose an item.

Statistics

Are you able to assess all statistics in the manuscript, including the appropriateness of statistical tests used? Choose an item.

Quality of Written English

Please indicate the quality of language in the manuscript: Choose an item.

Declaration of Competing Interests

Please complete a declaration of competing interests, considering the following questions:

- Have you in the past five years received reimbursements, fees, funding, or salary from an organisation that may in any way gain or lose financially from the publication of this manuscript, either now or in the future?
- Do you hold any stocks or shares in an organisation that may in any way gain or lose financially from the publication of this manuscript, either now or in the future?
- Do you hold or are you currently applying for any patents relating to the content of the manuscript?
- Have you received reimbursements, fees, funding, or salary from an organization that holds or has applied for patents relating to the content of the manuscript?
- Do you have any other financial competing interests?
- Do you have any non-financial competing interests in relation to this paper?

If you can answer no to all of the above, write 'I declare that I have no competing interests' below. If your reply is yes to any, please give details below.

I work for GigaScience where I perform checks on the quality of image data and the image analysis approach. My funding is not dependent on the outcome of this review, and I declare that I have no competing interests.

I agree to the open peer review policy of the journal. I understand that my name will be included on my report to the authors and, if the manuscript is accepted for publication, my named report including any attachments I upload will be posted on the website along with the authors' responses. I agree for my report to be made available under an Open Access Creative Commons CC-BY license (http://creativecommons.org/licenses/by/4.0/). I understand that any comments which I do not wish to be included in my named report can be included as confidential comments to the editors, which will not be published.

Choose an item.

To further support our reviewers, we have joined with Publons, where you can gain additional credit to further highlight your hard work (see: https://publons.com/journal/530/gigascience). On publication of this paper, your review will be automatically added to Publons, you can then choose whether or not to claim your Publons credit. I understand this statement.

Yes Choose an item.
