# Supplementary material for: ChronoRoot: High-throughput phenotyping by deep segmentation networks reveals novel temporal parameters of plant root system architecture
Source: Gigascience. 2021 Jul 20;10(7):giab052. doi: 10.1093/gigascience/giab052 (PMC8290196; doi:10.1093/gigascience/giab052)
Supplement: giab052_Reviewer_3_Report_Original_Submission — Valerio Giuffrida -- 3/10/2021 Reviewed [file giab052_reviewer_3_report_original_submission.pdf]

Reviewer Report

Title: ChronoRoot: High-throughput phenotyping by deep segmentation networks reveals novel temporal parameters of plant root system architecture

Version: Original Submission **Date:** 3/10/2021

Reviewer name: Valerio Giuffrida

Reviewer Comments to Author:

This manuscript presents a hardware+software framework for root analysis empowered by deep learning algorithms. The paper is very well written, detailed, and interesting to both computer vision and plant biologist communities.
I have several minor comments that are likely to be easily addressable by the authors, and one (potential) major comment.
Minor comments:
- Dataset: the authors give a lot of details re the dataset used for the deep learning models. However, these details are machine learning focused (e.g., we have X videos, we annotated Y images, etc.). It's unclear how many plants were used to generate such time series
- Training/Val set & Experimental dataset: the authors have two datasets: one used for training and validating the models, one to actually applied the trained model in a real-case scenario. Could you please state if these datasets are disjoined (e.g., a plant appearing in the training DOES NOT appear in the other dataset and vice versa). [*]
- temporal consistency refinement: as far as I understood from page 10, this step is performed as follows: I take an image at time t and one at time t-1 and I average them. Clearly, the root has grown in between. My question here is: how can you ensure that, by applying this method, you are not also getting rid of new grown material at time t that were not at t-1? Can it happen that you actually remove true positive that were not in the previous frame?
- Analysis section:
---- "Our model takes a sequence of images as input and outputs a labeled graph for each frame" I don't think this statement is correct. In my mind (and also according to what the authors meant), a model is referred to the deep learning models, right? As such, the output of such models is not a labeled graph, but it's a segmentation mask. Did the author mean to say "Our framework takes a sequence [...]"?
---- References to state-of-the-art methods (e.g., unet) are missing in this section
---- Results of table 1 comes before the authors introduce what CRF is and what it's for. In fact, I looked at the table as I was reading the paper and I was confused by the presence of Table 1. I suggest the authors to present the results AFTER they have introduced all the things contained in table 1.
---- "As shown in Figure 1, we apply several post-processing steps after segmentation" I don't think Fig 1 actually shows any post-processing. After step 4 (deep net), there is ROI selection and multi-class labeling
- Fig 5(c): Frequency is misspelled
- I am not a plant biologist and I apologise in advance if my doubt here sounds naive. At the end, I could

not understand what the reason behind the FFT analysis. At the end, what is this analysis telling me (please explain this in lay terms as I hardly got the message written in the text).
- CRF: on page 9, the authors say the parameters for the CRF are theta=5 and theta=3. Is it correct that the same parameter has two values?
- When the authors derive the graph representation of the plants, how do they deal with discontinuities arising from the segmentation process? In my experience, some times lateral roots exhibit discontinuity near the branching point.
- The authors used a dicot plant (arabidopsis thaliana). Could please the authors comment (only here, they don't need to change the main paper to answer to this question) how their work would apply for monocot (e.g., barley) plants? In my experience, lateral roots of barley are rather thin and hard to be segmented.
Major point:
Picking up my second minor comment (the one marked with a [*]), I have this doubt. During the training, it is not clear how the dataset was split between training/ validation (if used)/test set. Are they treating each image individually? Or they are treating each time series (videos as they call) individually? This is important to be clarified for the following reasons:
- Images treated individually: in this case, it can happen that the image of a plant at time t can appear in the training set, while another frame of the same plant (let's say t+2) happens to be in the testing set. If this is the case, I think this is wrong because the testing set contains images that the network has already seen (although at a different development stage). In my personal experience, I've got rather biased (too good to be true!) results when images of a time series are used independently to each other.
- Time series used individually: if n time series are used for training and m for testing (and they are disjoined), then this is the correct procedure.
Therefore, I ask the authors to clarify which of the two protocols they used. In the case they treated each image independently, I highly recommend to run all the training and testing again treating each time series as a whole.

Methods

Are the methods appropriate to the aims of the study, are they well described, and are necessary controls included? Choose an item.

Conclusions

Are the conclusions adequately supported by the data shown? Choose an item.

Reporting Standards

Does the manuscript adhere to the journal’s guidelines on minimum standards of reporting? Choose an item.

Choose an item.

Statistics

Are you able to assess all statistics in the manuscript, including the appropriateness of statistical tests used? Choose an item.

Quality of Written English

Please indicate the quality of language in the manuscript: Choose an item.

Declaration of Competing Interests

Please complete a declaration of competing interests, considering the following questions:

- Have you in the past five years received reimbursements, fees, funding, or salary from an organisation that may in any way gain or lose financially from the publication of this manuscript, either now or in the future?
- Do you hold any stocks or shares in an organisation that may in any way gain or lose financially from the publication of this manuscript, either now or in the future?
- Do you hold or are you currently applying for any patents relating to the content of the manuscript?
- Have you received reimbursements, fees, funding, or salary from an organization that holds or has applied for patents relating to the content of the manuscript?
- Do you have any other financial competing interests?
- Do you have any non-financial competing interests in relation to this paper?

If you can answer no to all of the above, write 'I declare that I have no competing interests' below. If your reply is yes to any, please give details below.

I declare that I have no competing interests

I agree to the open peer review policy of the journal. I understand that my name will be included on my report to the authors and, if the manuscript is accepted for publication, my named report including any attachments I upload will be posted on the website along with the authors' responses. I agree for my report to be made available under an Open Access Creative Commons CC-BY license (http://creativecommons.org/licenses/by/4.0/). I understand that any comments which I do not wish to be included in my named report can be included as confidential comments to the editors, which will not be published.

Choose an item.

To further support our reviewers, we have joined with Publons, where you can gain additional credit to further highlight your hard work (see: https://publons.com/journal/530/gigascience). On publication of this paper, your review will be automatically added to Publons, you can then choose whether or not to claim your Publons credit. I understand this statement.

Yes Choose an item.
